# Incidence and impact of other malignancies after immunochemotherapy by fludarabine, cyclophosphamide, and rituximab as frontline treatment for chronic lymphocytic leukemia: A single-center retrospective study.

**DOI:** 10.46989/001c.127828

**Published:** 2025-01-03

**Authors:** Nicolas Stocker, Tamim Alsuliman, Elise Corre, Laure Ricard, Fazia Kaoui, Paul Coppo, Eolia Brissot, Remy Dulery, Anne Banet, Zoé Van de Wyngaert, Ollivier Legrand, Agnès Bonnin, Mohamad Mohty, Florent Malard, Zora Marjanovic

**Affiliations:** 1 Service d’Hématologie Clinique et Thérapie Cellulaire Hôpital Saint-Antoine; 2 Centre de Recherche Saint-Antoine Sorbonne Université

**Keywords:** Chronic lymphocytic leukemia, Immunochemotherapy, Second malignancy

## Abstract

Individuals with chronic lymphocytic leukemia (CLL) or small lymphocytic lymphoma (SLL) have a high risk of developing other malignancies (OMs). The development of OMs may be associated with the advanced age of CLL/SLL patients, presence of a tumor-promoting microenvironment, immune alterations inherent to CLL/SLL, or chemotherapy. Importantly, the occurrence of OMs following frontline fludarabine, cyclophosphamide and rituximab (FCR) treatment is associated with a reduction in the overall survival (OS). This retrospective study included 108 CLL/SLL patients treated with FCR immunochemotherapy, as a first line treatment. With a median follow-up of 94.9 (6-222) months, 31% developed an OM or more, within a median of 61.8 months post-FCR initiation. The most common OMs were non-melanoma skin cancers (7%), Richter’s syndrome (RS) (7%), myelodysplastic syndromes (6%), prostate cancer (4%), and acute myeloid leukemia (3%). Patients with OMs had shorter survival compared to those without (104.0 versus 149.0 months, P=0.02), with RS having the worst OS at 4.8 months (P<0.0001), followed by therapy-related myeloid neoplasia (t-MN) at 14.5 months. Although the onset of OMs in patients with CLL/SLL was observed after considerable delays, its impact on survival is significant in the immunochemotherapy era, necessitating a better understanding of these patterns to improve CLL/SLL management and guide future treatment strategies.

## Introduction

Chronic lymphocytic leukemia (CLL) is the most common type of leukemia in the Western world.^1^ During the period of gradual progression, which may extend to several years, individuals may not require treatment, allowing them to lead a satisfactory life despite their condition.

Furthermore, individuals with CLL/small lymphocytic lymphoma (SLL) frequently face substantial health issues stemming from infections, autoimmune disorders, and an increased risk of both solid and hematologic cancers, compared with the general population.^2–4^ In particular, fludarabine, cyclophosphamide, and rituximab (FCR) immunochemotherapy, which was historically a widely used frontline treatment for physically fit patients,^5^ has been linked to an increased risk of developing other malignancies (OMs).^6–9^ Studies have demonstrated a noteworthy increase in the incidences of acute myeloid leukemia (AML) and myelodysplastic syndrome (MDS).^10–13^ The precise mechanisms contributing to the development of OMs may be associated with individual patient-specific risk factors, notably the advanced age of CLL/SLL patients,^9^ presence of a tumor-promoting microenvironment, immune alterations inherent to CLL/SLL,^11^ or chemotherapy.^5,14^ Importantly, the occurrence of OMs following frontline FCR treatment is associated with a reduction in the overall survival (OS) of these individuals.^7,11,12^

Several studies have documented the leukemogenic potential of the purine analog fludarabine, particularly when combined with other DNA-damaging agents, such as cyclophosphamide. Fludarabine impedes DNA repair and enhances the cytotoxic effects of these drugs. This heightened DNA damage can affect bone marrow progenitor cells, resulting in prolonged myelosuppression and diminished immune surveillance. Consequently, there is a greater likelihood of developing therapy-related myeloid neoplasia (t-MN) with these drugs, as has been reported.^7,15^ Furthermore, rituximab, commonly prescribed for CLL/SLL, can cause extended neutropenia in 30–52% of treated patients.^5^ Prolonged rituximab exposure may also trigger B-cell depletion, further compromising immune surveillance and increasing the likelihood of OM development. While it appears that recent advancements in the treatment of CLL/SLL have shifted towards the use of chemotherapy-free medications, the use of cytotoxic drugs has significantly decreased, suggesting a substantial reduction in the risk of t-MN development.

This study aimed to assess the incidence, describe the characteristics of OMs, and analyze their impact on the outcomes of patients with CLL/SLL who received frontline FCR immunochemotherapy.

## Materials and Methods

### Patients

This retrospective study was conducted in a cohort of CLL/SLL patients who were treated with frontline FCR immunochemotherapy at Saint-Antoine University Hospital (AP-HP, Paris, France), between November 2005 and April 2023. All patients were diagnosed according to the criteria set by the International Workshop on CLL (IWCLL), and were deemed by their treating clinician to require treatment, due to symptoms (systemic signs, rapid progression of lymphocytosis or tumor syndrome) or low hemoglobin or platelets, or bone marrow infiltration by CLL cells, classified as Binet stage C. The purpose of this study was to document the occurrence of OMs and assess their impact on outcomes in patients with CLL/SLL.

### FCR immunochemotherapy

The patients underwent 4-6 cycles of immunochemotherapy with fludarabine administered at dosages ranging from 25 to 30 mg/m^2^, and cyclophosphamide administered at dosages ranging from 250 to 300 mg/m^2^ on days 1 to 3, throughout cycles 1 to 6. Rituximab was administered on day 1 at a dose of 375 mg/m^2^ in the first cycle and 500 mg/m^2^ in cycles 2–6.

### Other malignancies

The scope of this study encompasses all solid and hematological malignancies. Our examination focused on the initial occurrence of OMs, excluding those diagnosed before the start of the follow-up period.

### Statistical analysis

The summary of each continuous variable is expressed by the median and range, while the summary of each categorical variable is provided in terms of frequency and percentage. To test for differences between the risk groups, the Wilcoxon rank-sum test was used for continuous data and the Fisher’s exact test for categorical data. Progressive disease was defined according to the criteria set by the IWCLL. Progression-free survival (PFS) was defined as the duration from the start of treatment to the occurrence of progressive disease, initiation of salvage therapy, or death, whichever occurred first. OS was calculated from the start of treatment to the date of death, or the last observation if the patient remained alive. PFS and OS probabilities were determined using the Kaplan-Meier method, and comparisons were made using the log-rank test. The significance threshold (Type I error rate) was set to 0.05. GraphPad Prism 10 and EZR version 1.62 (Saitama Medical Center, Jichi Medical University, Japan) were used for statistical analysis and graphical representation, with the latter serving as a graphical interface for R (The R Foundation for Statistical Computing; version 4.3.2).

## Results

### Patient and treatment characteristics

**Table 1** presents an overview of the pretreatment features of the entire study population (n=108). The median age was 63 years (range: 37–82), and the majority (64%) of the participants were male. The diagnostic categories included CLL (91%) and SLL (9%). The median follow-up period for the 108 patients after FCR immunotherapy was 94.9 months (range, 6-222). Fifty-eight percent of the patients were classified as Binet stage B, 24% as stage C, and 18% as stage A, having received treatment because of notable constitutional symptoms and/or rapid lymphocyte doubling time. According to conventional karyotyping and fluorescence *in situ* hybridization (FISH) analyses, 20% of the patients displayed trisomy 12, 15% had deletions in 11q, 9% exhibited deletions in 13q, and 7% showed structural alterations resulting in the loss of *TP53* (deletion in 17p or *TP53* mutation). The *IGHV* genetic status was mutated (*IGHV*-M) in 7% of the patients, unmutated (*IGHV*-UM) in 11%, and unknown in 82% of patients because of insufficient pretreatment material. The median time from diagnosis was 31.5 months (range, 0-268), with the majority of patients undergoing six cycles of FCR (57%), 26% receiving four cycles, and 17% completing fewer than four cycles of treatment.

**Table 1.** Patient characteristics

Characteristic	N=108
Age, years (range)	
Median	63 (37-82)
≥ 70 years	74 (70-82)
Gender, n (%)	
Male	69 (64)
Female	39 (36)
Diagnosis, n (%)	
Chronic lymphocytic leukemia	98 (91)
Small lymphocytic lymphoma	10 (9)
Binet stage, n (%)	
A	19 (18)
B	63 (58)
C	26 (24)
Cytogenetic subgroups, n (%)	
Trisomy in 12	22 (20)
Deletion in 11q	16 (15)
Deletion in 13q	10 (9)
Deletion in 17p or *TP53* mutation	8 (7)
Complex (≥3 abnormalities)	5 (5)
No abnormalities	20 (19)
Could not be evaluated	24 (22)
*IGHV* mutational status, n (%)	
Mutated	8 (7)
Unmutated	12 (11)
Could not be evaluated	88 (82)
Serum β2microglobulin, n (%)	
Normal	12 (11)
Elevated	7 (7)
Could not be evaluated	89 (82)
FCR treatment, n (%)	
< 4 cycles	18 (17)
4 cycles	28 (26)
6 cycles	62 (57)
Median time from diagnosis, months (range)	31.5 (0-268)

FCR=fludarabine, cyclophosphamide and rituximab; *IGHV*=immunoglobulin heavy-chain variable region gene; n=number.

### Risk of OM among CLL/SLL patients

**Table 2** shows the prevalence of hematological and non-hematological OMs. After a median follow-up period of 94.9 months, 34 (31%) patients were diagnosed with OMs, with a median time to OM onset of 61.8 months (range, 2-181) from the initiation of FCR therapy. The median time interval from the diagnosis of CLL/SLL to the onset of OM was 99.2 months (range, 3-329). The primary hematological OM distribution included RS (7%), MDS (6%), AML (3%), and acute lymphocytic leukemia (ALL, 1%) (Supplementary Figure S1). Non-hematological OMs comprised non-melanoma skin cancer (NMSC, 7%), cancer of the prostate (4%), lung (3%), breast (2%) and colon (1%) (Supplementary Figure S1). The incidence of OMs among older patients (those above the median age) was 33% and there were no differences between patients who received ≤4 or 6 cycles of FCR immunochemotherapy (35% and 29%, respectively). Among patients who developed OMs, the median time to the occurrence of t-MN was 82.8 months (range, 28-181), compared to 59.2 months (range, 9-159) for non-hematological OMs and 84.1 months (range, 2-112) for RS.

**Table 2.** Characteristics of other malignancies

Characteristics	N=108
Type of OM, n (%)	
Hematological	17 (16)
Non-hematological	17 (16)
OM incidence above the median age, n (%)	16 (33)
OM incidence according to FCR treatment, n (%)	
≤4 cycles	16 (35)
6 cycles	18 (29)
Hematological OM, n (%)	
Richter’s syndrome	7 (7)
Myelodysplastic syndrome	6 (6)
Acute myeloid leukemia	3 (3)
Acute lymphoid leukemia	1 (1)
Non-hematological OM, n (%)	
Non-melanoma skin cancers	7 (7)
Prostate cancer	4 (4)
Lung cancer	3 (3)
Breast cancer	2 (2)
Colon cancer	1 (1)
Median time to OM onset from diagnosis, months (range)	99.2 (3-329)
Median time to OM onset after FCR treatment, months (range)	61.8 (2-181)
Richter’s syndrome	84.1 (2-112)
Therapy-related myeloid neoplasia	82.8 (28-181)
Non-hematological	59.2 (9-159)

OM=other malignancy; FCR=fludarabine, cyclophosphamide and rituximab; n=number.

### Impact of OM occurrence on CLL/SLL survival

The clinical outcome characteristics of the CLL/SLL patients are summarized in **Table 3**.

**Table 3.** Clinical outcomes after FCR treatment

Outcomes	N=108	*P*-value
Median follow-up, months (range)	94.9 (6-222)	
Progression-free survival, months (95%CI)	64.5 (54-78)	
Time to next treatment, months (95%CI)	79.0 (61-123)	
Overall survival, months (95%CI)	120.0 (102-166)	
Overall survival according to OM occurrence, months (95%CI)		
With OM	104.0 (85-111)	**0.02**
Without OM	149.0 (113-NA)	
Overall survival according to OM type, months (95%CI)		
Hematological	89.4 (48-120)	0.17
Non-hematological	109.6 (85-NA)	
Median overall survival following OM diagnosis, months (95%CI)		
Richter’s syndrome	4.8 (0-9)	**<0.0001**
Therapy-related myeloid neoplasia	14.5 (0-NA)	
Non-hematological	41.4 (19-NA)	

OM=other malignancy; FCR=fludarabine, cyclophosphamide and rituximab; CI=confidence interval.

**Figure 1.**
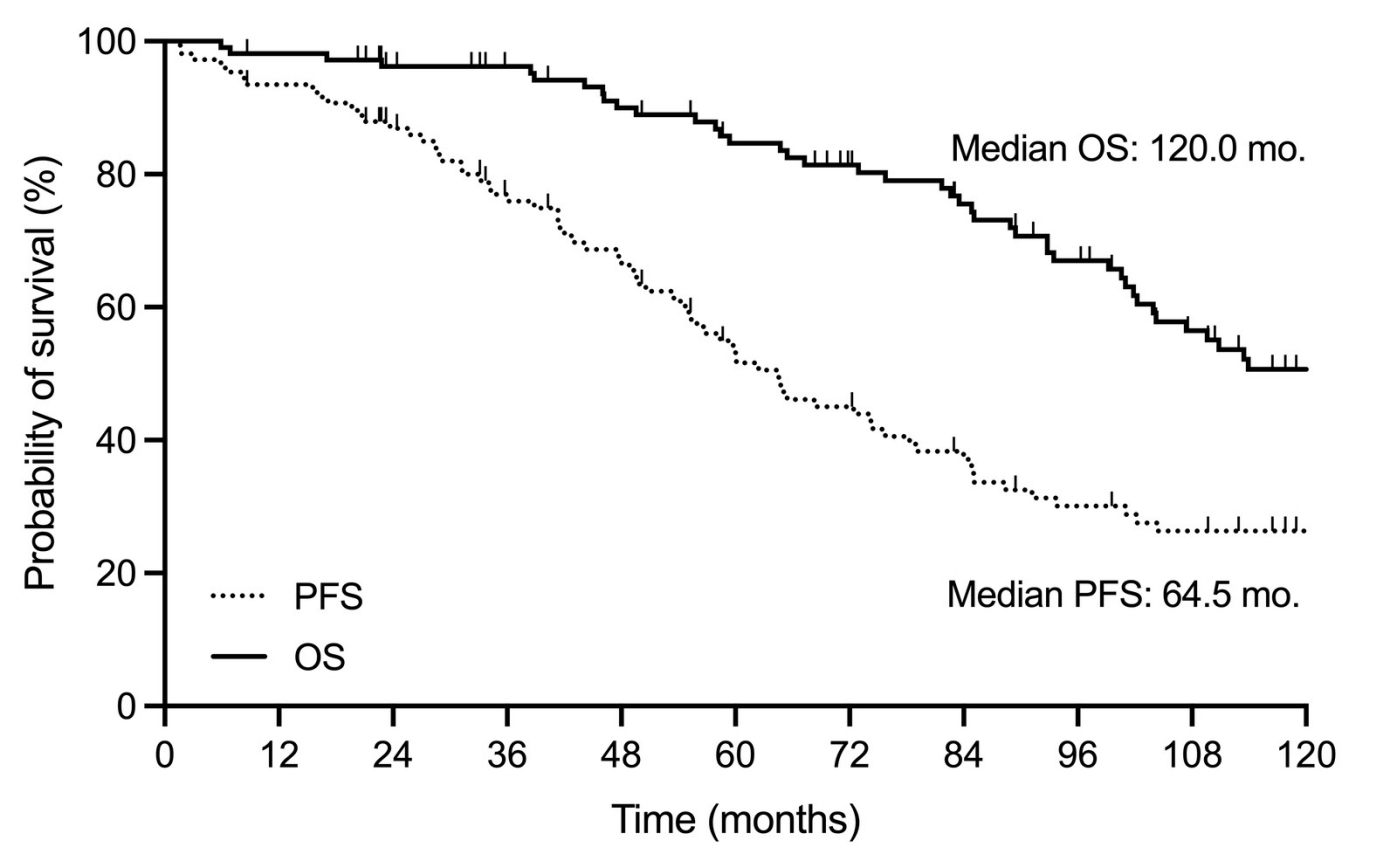
Probability of survival following frontline fludarabine, cyclophosphamide and rituximab immunochemotherapy. Progression-free survival and overall survival in the study population. FCR, fludarabine, cyclophosphamide and rituximab

**Figure 2.**
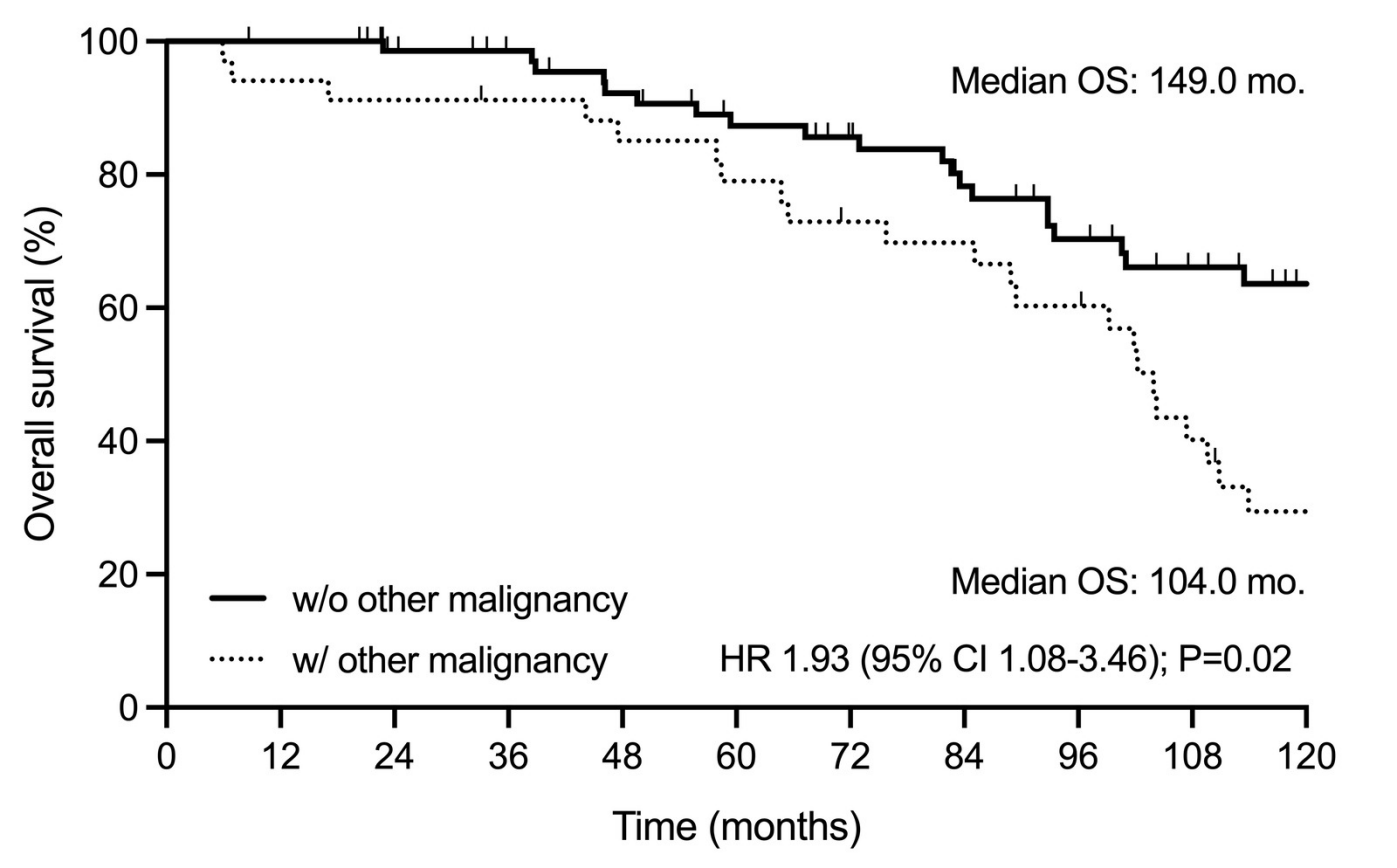
Overall survival according to the occurrence of other malignancy. Overall survival of patients with CLL/SLL with or without an OM diagnosis. CLL/SLL, chronic lymphocytic leukemia/small lymphocytic lymphoma; OM, other malignancy.

**Figure 3.**
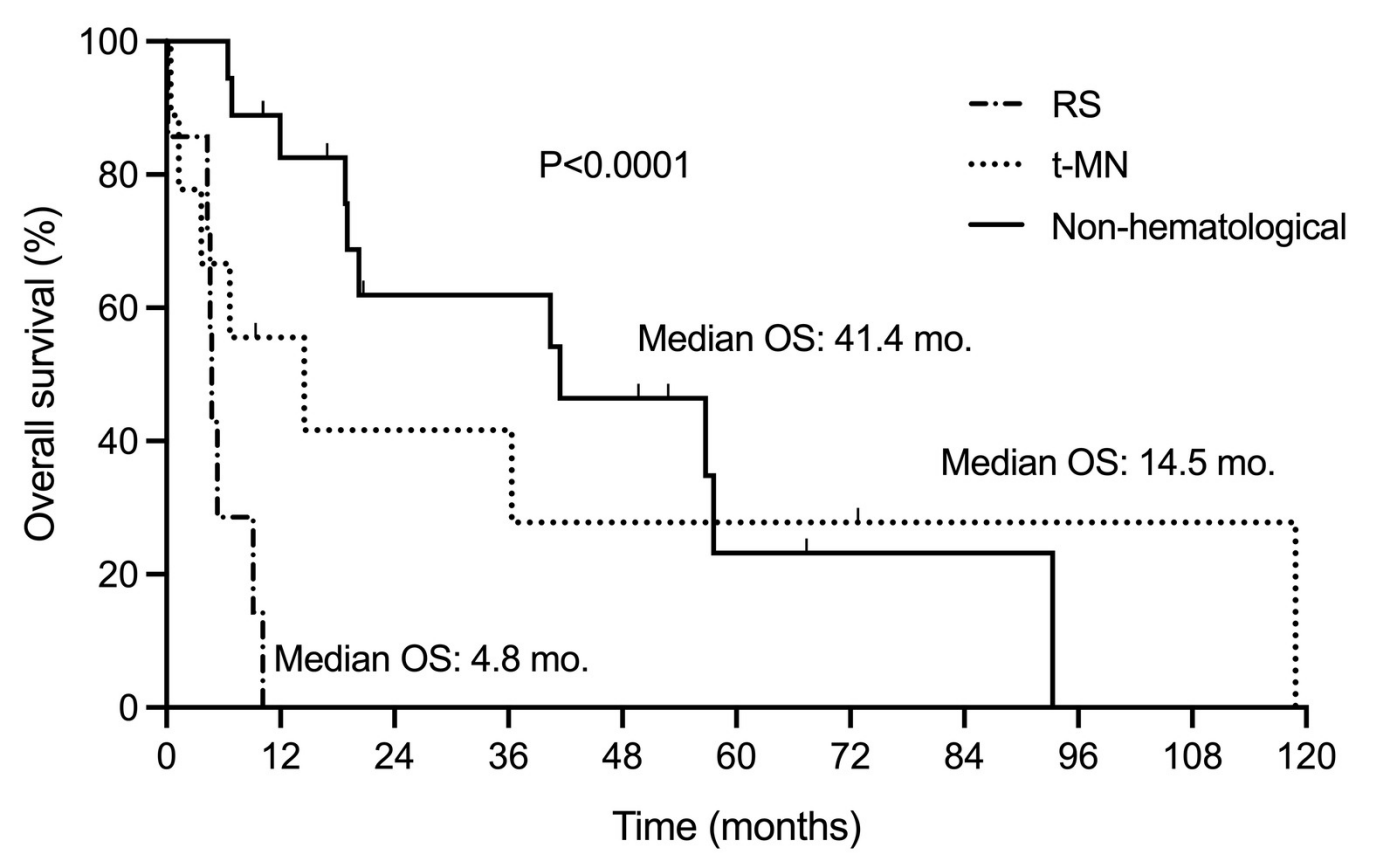
Overall survival following diagnosis of other malignancy. Overall survival of CLL/SLL patients with Richter’s transformation, therapy-related myeloid neoplasia, and other OMs. CLL/SLL, chronic lymphocytic leukemia/small lymphocytic lymphoma; OM, other malignancy; RS, Richter’s syndrome; t-MN, therapy-related myeloid neoplasm.

The median PFS was 64.5 months (95% confidence interval [CI], 54-78), and the median OS was 120.0 months (95%CI, 102-166), as depicted in **Figure 1**. The median time to next treatment was 79.0 months (95%CI, 61-123) (**Supplementary Figure S2**). Patients with OMs demonstrated significantly shorter survival following FCR immunochemotherapy than those without OMs (104.0 versus 149.0 months, P=0.02) (**Figure 2**). There was no significant difference in OS between patients with hematological and non-hematological OMs (89.4 versus 109.6 months, P=0.17) (**Supplementary Figure S3**). There was a significant difference (P<0.0001) in OS following the diagnosis of OMs. The median OS from RS diagnosis was 4.8 months (95%CI, 0-9), being 14.5 months (95%CI, 0-NA) for t-MN patients and 41.4 months (95%CI, 19-NA) for patients with non-hematological OMs (**Figure 3**).

## Discussion

Patients with CLL/SLL have an increased risk of developing an OM, which is one of the leading causes of death in these individuals.^2^ However, treatment of CLL/SLL and other cancers (hematological or non-hematological), with a combination of alkylating agents and purine analogs, has been shown to predispose to the emergence of OMs in these patients.^11,12^

In this study, we assessed the incidence of OMs in a cohort of CLL/SLL patients who received frontline FCR immunotherapy. Several previous reports have shown that patients treated with FCR therapy had a risk of OM of up to 2.38 times higher than that of the general population,^2–4^ with significantly higher incidences of t-MN in particular. Thirty-one percent of patients in our study developed OMs following frontline FCR treatment, with a balanced distribution between hematological and non-hematological Oms, and a median time to onset of 61.8 months. t-MN was the primary etiology of OM in our study (6% MDS and 3% AML), followed by NMSC (7%), and RS (7%).

In a recently published study, diagnosis of OM was reported in 4134/19,705 (21%) patients with CLL; of these, 633 (3.2%) had RT or B-cell prolymphocytic leukemia (B-PLL). Most patients (15.7%) were diagnosed with one or more solid tumors, while 4.2% of patients had a second hematological malignancy, and 1% had both (Chatzikonstantinou, Scarfo et al., 2023). In our cohort the incidence rate was higher (31%), which may be explained by the longer follow-up, better data traceability (one-center cohort) and, eventually, by the differences in cytogenetic profiles (e.g. only 19% of our cohort exhibited normal karyotype).^16^

Several studies showed that the rate of t-MN approaches 4 to 5% following frontline FCR treatment^15^ These rates were higher than those previously reported following treatment with alkylating agents, purine analogs, or both.^17,18^ The addition of rituximab could also potentiate the myelotoxicity of FC-type immunochemotherapy, as suggested by the FCR3 protocol (rituximab dose three times higher than the standard FCR regimen).^7^ However, the incidence of t-MN did not increase in the FCR arm of the CLL8 study.^17^

Skin cancers, particularly malignant melanoma and basal cell carcinoma, are the most frequently observed type of OM in patients with CLL/SLL.^19,20^ The risks are multiplied by 3 and 14, respectively, in these patients.^3,14,21^ These skin cancers can exhibit increased aggressiveness, high recurrence rates, and more regional metastases compared to non-hematological cancers.^3,21–23^ Although the risk of skin cancer increases gradually with age, and is significantly higher in patients with CLL/SLL, FCR treatment could also contribute to the risk of developing NMSC. In our study, seven of 108 patients (7%) developed NMSC, concurring with published incidence rates.^19,20,23^

RS, classically defined as the development of an aggressive lymphoma from a clonally related malignant B-cell originating from the original CLL (80% of cases), occurred in 7 (7%) of our study patients, consistent with published incidence rates.^17,24–26^ The influence of purine analog treatment and its association with RS is not clear, and the evidence remains insufficient, due to small sample sizes and wide CIs. Several reports indicate a high incidence of RS in patients treated with fludarabine,^27^ with it approaching 6 to 7% in clinical trials.^17,26,28^ In an analysis using death as a competing risk, the hazard ratio for Richter’s transformation in treated compared to untreated patients was 2.18.^29^

Historically, patients experiencing an OM after frontline FCR treatment had significantly lower OS compared to patients without a secondary cancer, as previously described in the literature.^7,11,30^ Our results were qualitatively comparable with these data, showing 104 months versus 149 months of median OS for patients with other malignancies versus those without OMs, respectively, with a 0.02 p-value. The more quantitively pronounced difference may be explained by longer follow-up. Interestingly, no significant difference in survival between patients with hematological and non-hematological OMs was observed in our cohort. This study was limited by the failure to document the causes of death in some patients. This difficulty may be explained by the relatively long follow-up period, along with the wide geographic distribution of our cohort of patients.

While the causal role of FCR can be discussed in this context, the spontaneous transformation from chronic to acute leukemia, namely from CLL to AML, although rare, has been already described. It has been also showed that AML can occur secondarily to CLL under treatments other than FCR.^31,32^

However, it has been well demonstrated that secondary MDS and AML have a bleaker prognosis than *de novo* forms.^33,34^ Furthermore, it has been shown that patients with CLL/SLL have an increased risk of death due to malignant melanoma and to NMSC, with standard mortality ratios of 4.79 and 17.0, respectively. Finally, the prognosis of RS remains very poor, although the clonal relationship between RS and the underlying CLL/SLL may influence treatment response.^35^ In our study, RS was significantly associated with the poorest OS (median of 4.8 months).

Our analysis revealed OM rates comparable to those reported in real-world literature,^7,8,11,12^ traditionally higher than rates in clinical trials, partly due to longer observation periods and lack of inclusion criteria (e.g., younger age, absence of comorbidities). Although current treatment trends limited the chemotherapy indication in CLL, these results are important to better understand the risk factors of OMs in CLL, especially for patients historically treated with immunochemotherapy. However, this retrospective, chart review-based study had incomplete data which may have introduced bias.

With remarkable advancements in CLL/SLL treatment since the introduction of targeted therapies, the use of FCR has been declining. Furthermore, the high incidence of OMs and the associated excess mortality may represent an additional significant limitation to the future use of chemotherapy for this disease. The incidence of other malignancies in the context of treated CLL patients should be studied in randomized controlled trials in order to identify the relative risk associated with every treatment modality.

### Authors’ Contribution

Conceptualization: Stocker, Mohty, Malard and Marjanovic; Data curation: Stocker; Formal Analysis and Investigation: Stocker, Alsuliman and Marjanovic; Methodology: Stocker, Mohty, Malard and Marjanovic; Writing – original draft: Stocker and Alsuliman; Writing – review & editing: all authors.

### Competing Interests – COPE

NS reports lecture honoraria from AbbVie, Astra Zeneca, and Janssen, all outside of the submitted work. FM reports lecture honoraria from Therakos/Mallinckrodt, Janssen, Biocodex, Sanofi, Jazz Pharmaceuticals, Gilead, and Astellas, all outside of the submitted work. TA reports lecture and/or consulting honoraria from Biotest and Amgen, all outside of the submitted work. MM reports grants and lecture honoraria from Janssen, Sanofi, Maat Pharma, and JAZZ Pharmaceuticals; lecture honoraria from Celgene, Amgen, BMS, Takeda, and Pfizer; and grants from Roche, all outside the submitted work. EB reports lecture honoraria from Novartis, Astellas, Alexion, Jazz Pharmaceuticals, Gilead/Kyte, MSD, Keocyt, Amgen, Pierre Fabre, BeiGene, all outside the submitted work. The other authors declare no competing financial interests.

### Ethical Conduct Approval – Helsinki – IACUC

All patients provided written informed consent to participate in the study.

### Informed Consent Statement

All authors and institutions have confirmed this manuscript for publication.

### Data Availability Statement

Requests from external parties for data will be considered on a case- by-case basis. The authors reserve the right to deny requests for all appropriate reasons. Data requests that risk sharing participant-level data or proprietary information will not be approved.
